# The Use of Arts‐Based Methods to Enhance Patient Engagement in Health Research

**DOI:** 10.1111/hex.70127

**Published:** 2024-12-16

**Authors:** Emily K. Phillips, Anna M. Chudyk, Caroline Monnin, Annette S. H. Schultz, Rakesh C. Arora, Todd A. Duhamel, Sheila O'Keefe‐McCarthy

**Affiliations:** ^1^ Applied Health Sciences, Faculty of Graduate Studies University of Manitoba Winnipeg Manitoba Canada; ^2^ College of Pharmacy University of Manitoba Winnipeg Manitoba Canada; ^3^ Neil John Maclean Health Sciences Library University of Manitoba Winnipeg Manitoba Canada; ^4^ College of Nursing, Rady Faculty of Health Sciences University of Manitoba Winnipeg Manitoba Canada; ^5^ Institute of Cardiovascular Sciences St. Boniface Hospital Albrechtsen Research Centre Winnipeg MB Canada; ^6^ Harrington Heart and Vascular Institute University Hospitals – Cleveland Medical Center/Case Western Reserve University Cleveland Ohio USA; ^7^ Faculty of Kinesiology and Recreation Management University of Manitoba Winnipeg Manitoba Canada; ^8^ Department of Nursing, Faculty of Applied Health Sciences Brock University St. Catharines Ontario Canada

**Keywords:** arts‐based research methods, patient and public involvement, patient engagement in research, patient involvement and engagement, patient‐oriented research, photography

## Abstract

**Introduction:**

Patient and care partner engagement in research (PER) is important in generating knowledge to improve healthcare. Arts‐based methods (ABM) use art in the research process to share aesthetic knowledge, which is knowledge that may be too complex to share only verbally. Together, PER and ABM are potentially synergistic, as both are participatory, problem‐focused, dialogic, and collaborative; yet little is known of the utility of ABM for PER.

**Methods:**

A narrative review was performed to identify, collate, and summarize the ways ABM has been used with PER and share the impacts of ABM on PER. The databases CINAHL, Scopus, and PubMed were searched, and 15 articles were included.

**Results:**

A wide variety of ABM were used for PER, with some studies using multiple ABMs. The use of ABM for PER was reported to be decolonizing, shifted power from researchers to people with lived experience, and reduced tokenism. People with lived experience shared their knowledge directly through their art, deepening the understanding of their emotions, feelings, and relationships.

**Conclusion:**

Researchers should consider the benefits of the participatory nature of ABM and explore how to engage people with lived experience in their work beyond data collection. Researchers engaging people with lived experience should consider using ABM as a way to operationalize PER to elicit aesthetic knowledge and strengthen power equalization.

**Patient or Public Contribution:**

No patients or members of the public contributed to this review due to a lack of funding to support their meaningful involvement.

Patient and care partner engagement in research (PER) refers to meaningfully and actively involving people with lived experience (PWLE) of the health issue being studied throughout the research process [1, 2, 3]. It is internationally recognized that PER in health research is important in generating knowledge to improve the healthcare system [1, 2, 3]. PER is feasible [4] and beneficial, as PWLE provides insights unique to the health issue being studied from those of researchers and clinicians [5, 6, 7]. These insights can be sought at every level, in every stage of the research process, and through many methods [8]. For example, PWLE can collaborate on identifying and prioritizing research topics through journey mapping [8]. An advisory group of PWLE can be consulted on data collection methods [8]. PWLE can direct dissemination through conference presentations and webinars [4, 8]. PER can be used to broaden the reach of integrated and end‐of‐grant knowledge translation through the connections of PWLE and with plain language dissemination [9]. The methods of PER are dominated by verbal communication [8], privileging knowledge that can be verbalized and excluding that which cannot. These dominant verbal methods may also exclude some patient groups who find it difficult to verbalize information, such as young people, people with learning disabilities, and those with lower levels of education. These already marginalized groups face further exclusion due to the lack of inclusive methods for PER.

## 1 Arts‐Based Research Methods

Arts‐based research methods (ABMs) use art to delve into aesthetic knowledge; defined as complex experiential knowledge beyond a language focus that includes expressions and emotions [10, 11]. Both evocative and provocative, ABM elicits emotion and feeling along with empirical knowledge [10]. Art can have multiple meanings [10], leading to richer dialogue and a deeper understanding of the issues being discussed. ABM can be used throughout the research process for data collection, analysis, and dissemination [10]. A variety of ABM is available for use, including photo‐elicitation [10, 12, 13, 14], poetry [10, 14, 15], theatre [10, 14, 16], dance [10, 17], and visual art [10]. Specifically for knowledge translation, ABM is evocative tools that elicit strong images, memories, or feelings [15] to translate complicated quantitative and qualitative data to varied academic and general audiences and their use is supported by the Canadian Institutes for Health Research (CIHR) [9].

## 2 Patient Engagement and Arts‐Based Research Methods

Currently, most PER is missing the aesthetic knowledge elicited by ABM [11]. There is an opportunity to leverage the synergy of ABM and PER. ABMs are inherently participatory [10, 18], making use of PER logic. Both ABM and PER are problem‐focused rather than restrained by philosophical rules that must be adhered to [10, 19]. Integral to PER is dialogue, where PWLE share and reflect on their experiences [19]. ABM is a mechanism to facilitate rich dialogue and help build relationships among diverse team members [10]. This relationship‐building can help to balance the power between researchers and PWLE, which is integral to PER [19]. Power is shifted to PWLE as they can use ABM to reciprocally share their knowledge and insights in the manner they wish [10].

The dominance of the use of verbal or written expression for PER [8] limits the knowledge shared to that which can be verbalized. There are a vast number of ABMs available for use for PER to elicit aesthetic knowledge, however, the current state of use of ABMs for PER is not known. A systematic review from 2011 explored the use of ABM in health research; however, not with PER [20]. A 2023 systematic review explored the use of ABM with youth with complex psychosocial needs [21]. While these authors discuss how ABMs have been used in this specific context with a particular population sub‐group and at different research stages, there is further scope to explore ABM use more broadly in PER. The purpose of this narrative review was to explore the ways ABM has been used for PER to understand the current state of this potentially synergistic relationship.

## 3 Methods

A narrative review was conducted as this type of review is used to identify, collate, and summarize literature [22, 23]. This aligned with the research question, which was what is the current state of the use of ABM for PER in existing published literature? Reporting of the search strategy for this narrative review was guided by the Preferred Reporting Items for Systematic Reviews and Meta‐Analysis (PRISMA) extension for reporting literature searches [24] with deviations where appropriate to align with a narrative, rather than scoping, review. The databases CINAHL with Full Text (EBSCOhost), Scopus (Elsevier), and PubMed (NIH) were searched simultaneously in July 2022 and updated in October 2024 with an iterative search strategy to identify appropriate search terms and techniques. These databases were selected as they cover research from a variety of health disciplines. The final search included the following broad search terms and Boolean operators: ‘*patient engagement*’ OR ‘*participatory*’ AND ‘*arts‐based*’. Limits and restrictions applied were published articles that were available in English [25]. There was no date limit set. The search was not peer‐reviewed. One reviewer (E.K.P.) completed title and abstract, followed by full‐text screening.

## 4 Results

**Figure 1:**
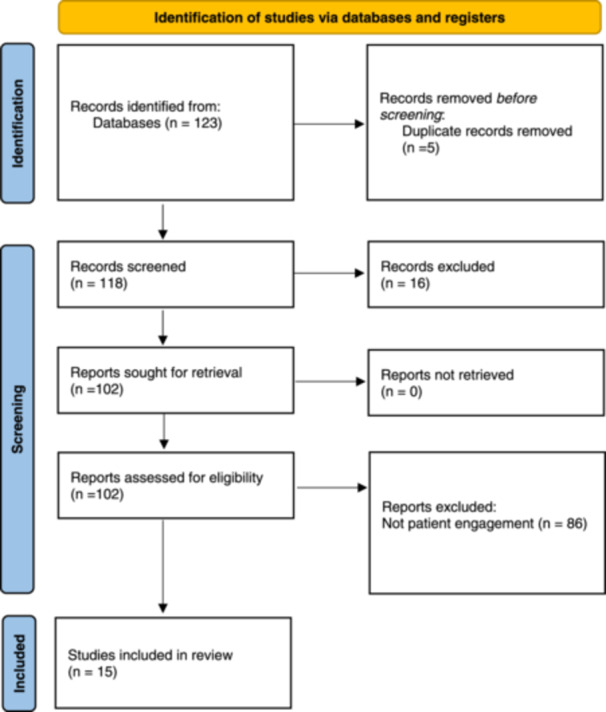
PRISMA diagram. ***Source***: M. J. Page, J. E. McKenzie, P. M. Bossuyt, et al., The PRISMA 2020 Statement: An Updated Guideline for Reporting Systematic Reviews. *BMJ* 372, no. 71 (2021). doi: 10.1136/bmj.n71.

A total of 123 articles were located. After removing five duplicates, the titles and abstracts of 118 articles were reviewed. Following this preliminary screening, 16 articles were excluded due to being focused on arts‐based therapies or education. All of the remaining 102 articles were available for full‐text review. Of these, 86 were excluded as they did not use ABM for PER but for data collection in studies that did not engage patients but rather studied them as research subjects in studies were the aim was not PER. One additional study was excluded as it was a review of reviews and did not provide the level of detail required for extraction. See Figure 1 for the PRISMA diagram.

### 4.1 Overview of Included Articles

**Figure 2:**
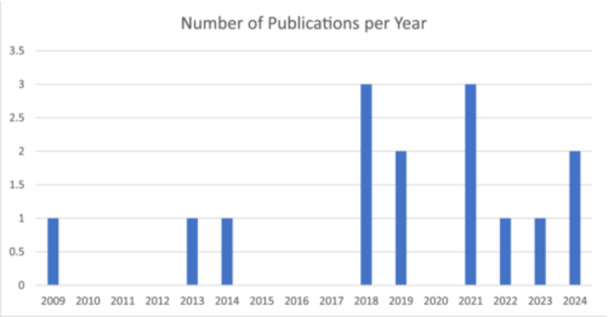
The number of included publications each year from 2009 to 2022 using ABM for PER.

An overview of the 15 included articles can be found in Table 1. Six of the studies were conducted in Canada [26, 27, 28, 29, 30], six in the United Kingdom [31, 32, 33, 34], two in the United States of America [35, 36], and one in Ireland [37]. As seen in Figure 2, the number of publications using ABM for PER has increased over time. The included articles engaged PWLE from across the lifespan, from paediatrics [26] to adults up to 75 years of age [34]. The majority of the studies (11) included PWLE who could be labelled as vulnerable, for example, adults with mental health issues [28, 30, 37], youth in care [29], disabled youth [33] and Indigenous peoples [27].

**Table 1:** Overview of included studies

Author & Year	Country	ABM Used	Age of participants	PER objectives	Level of engagement	Research stage	PER + ABM Impacts
Abousifein et al. [38]	Canada	Photovoice	60‐80	Plan an evaluation of engagement	Collaborate [41]	Identifying & Prioritizing [8] Research Design [8]	Sharing aesthetic knowledge
Archibald et al. [26]	Canada	Draw‐write‐tell	Youth (5–19) and adults	Identify knowledge exchange priorities	Consult [41]	Identifying & Prioritizing [8] Data Analysis [8]	n/a protocol
Ariss et al. [28]	Canada	Photovoice Poetry Visual art Songs/singing/music Photography	41 to 60+	Gain insights and input on methods and outcome measures	Consult [41]	Research Design [8] Data Collection [8] Data Analysis [8]	Perspectives Power
Casey and Webb [37]	Ireland	Photography Digital storytelling Visual art Theatre/improv/film/video	23 to 56	Explore personal meaning and perspectives of mental health recovery	Collaborate [41]	Data Collection [8] Data Analysis [8]	Power
Doucet et al. [29]	Canada	Photovoice	19 to 29	Gain perspectives of youth aging out of care	Collaborate [41]	Data Collection [8] Data Analysis [8] Dissemination [8]	Power
Hodges et al. [33]	UK	Poetry (performative)	14 to 20	Explore the nature of disability within society and their experiences of being disabled	Empower [41]	Data Collection [8] Dissemination [8]	Sharing aesthetic knowledge
Kelemen et al. [34]	UK	Cultural animation Poetry Music Theatre/improv/film/video	25 to 75	Explore what a healthy community is, ageing well and dying at home and how communities and government could collaborate to improve health	Collaborate [41]	Data Collection [8] Data Analysis [8]	Perspectives Power
Lauckner et al. [30]	Canada	Theatre/improv/film/video Visual art Spoken word Music	Not reported	Increase social inclusion through collaboration between the mental health and recreation sectors	Consult [41]	Research Design [8] Preparing for Execution [8] Data Collection [8] Dissemination [8]	Tokenism
Leteurtre et al. [40]	UK	Zines	Not reported	To co‐develop and inform a zine intervention and evaluation directed at pain‐related stigma	Collaborate [41]	Identifying & Prioritizing [8] Research Design [8] Data Collection [8]	Perceptions
Mackintosh et al. [32]	UK	Theatre/improv/film/video	25 to 45+	Develop and test film for knowledge translation to enable women seek professional help for serious safety concerns perinatally	Collaborate [41]	Data Collection [8] Data Analysis [8] Dissemination [8]	Sharing aesthetic knowledge
Rieger et al. [27]	Canada	Digital storytelling	Not reported	Explore use of a new research method	Collaborate [41]	Data Collection [8] Dissemination [8]	Decolonizing
Toye et al. [39]	UK	Poetry	Adolescents	Understand the experience of pain across conditions, contexts, and lifespan through a comprehensive review of qualitative research	Collaborate [41]	Data Analysis [8] Dissemination [8]	Perspectives
Tyler et al. [31]	UK	Visual art	Not reported	Understand quality and safety issues of healthcare for people living with for Vitamin B12 Deficiency or Pernicious Anaemia	Consult [41]	Identifying & Prioritizing [8] Data Collection [8] Dissemination [8]	Perspectives Power
Yonas et al. [36]	USA	Visual Voices	8–15	Understand perspectives on community safety	Collaborate [41]	Identifying & Prioritizing [8] Data Collection [8] Dissemination [8]	Perspectives Power
Yonas et al. [35]	USA	Visual Voices	8–15	Understand perceptions regarding adolescent health, safety, and violence	Collaborate [41]	Identifying & Prioritizing [8] Data Collection [8] Dissemination [8]	Perspectives Power

### 4.2 Arts‐Based Research Methods Employed

The articles included in this review used a variety of ABM for PER. Eleven articles employed one ABM, for example, Visual Voices [35, 36], photovoice [29, 38], film [32], digital storytelling [27], poetry [39], zine creation [40] and performative poetry [33]. Four articles employed multiple ABM for PER, including drama exercises, art making, poetry, music [34], theatre, visual art, spoken word, music [30], a variety of visual methods [37] and ﻿poems, songs, drawings, paintings, photographs, and a felted art piece [28]. See Table 1 for an overview of the types of ABM employed in each study.

### 4.3 Operationalization of ABM for PER

ABMs for PER were operationalized at the levels of consult, collaborate, and empower [41]. Examples of how consulting occurred are requesting input from participants [28] and engaging participants in priority setting [31]. Collaboration occurred through co‐developing the project with participants and including them in data analysis [35, 36] and co‐designing the research questions and analysing the research findings [34]. Empower was operationalized by having participants decide how to perform the poems they created [33]. These findings suggest that ABM can be used across the engagement spectrum from having less impact on decision‐ making (consult) to the most impact on decision‐making (empower) [41]. See Table 1 for details on levels of engagement.

While PER was operationalized across the research cycle [8], from priority setting [31], project advisement [30] and development [36], method and outcome measure selection [28], data analysis [32, 37], and knowledge translation [27, 29, 33, 35], ABMs for PER were operationalized for data collection with PWLE. See Table 1 for an overview of the research stages PER was operationalized at. For example, poetry was created by disabled youth to explore how to enhance public understanding and confront negative perceptions of disability [33]. Another study co‐created a script and storyboard for a video related to the experiences of seeking help for serious perinatal complications [32]. Regardless of the type of ABM used, a series of workshops where PWLE individually or collectively created art was commonly how ABM for PER were operationalized. For example, the cultural animation workshops explored the concept of healthy communities through three workshops exploring different questions: (1) ‘What is a healthy community?’ (2) ‘ageing well and dying at home’, and (3) ‘how communities and government could work together to improve health’ [34]. This format seems to support PWLE in focusing on a specific portion of their experience to allow them to share that experience deeply and holistically.

PWLE were often engaged in analysing the artistic data gathered. One study shared the five questions that guided their co‐analysis: (1) Describe your piece, (2) What is happening in your piece? (3) Why did you focus on this subject matter? (4) What does this artwork tell us about your recovery? (5) How can this artwork provide opportunities to support the recovery process? [28] The discussions related to the art were commonly audio recorded and transcribed and analysed thematically by the PWLE and researchers together [26, 28, 29, 32, 34].

Knowledge translation commonly included publicly sharing the art created [29, 30, 31, 33, 36]. This was either through existing events [30, 33] or through those planned specific to the research [29, 31, 36]. The video created in one study will be used clinically to support others navigating the same health issue, serious perinatal complications [32]. See Table 2 for descriptions of different types of ABM that were found to be used for PER and examples of how they have been operationalized.

**Table 2:** Overview of arts‐based methods used for patient engagement in research.

Type of ABM	Description of ABM	Example of how ABM can be used
Poetry [28, 33, 34, 39]	Poetry uses words, space, pauses, punctuation, and rhythm to convey meaning lyrically [10]. Poetry can be used to represent (a research method), interpret and understand experiences(data analysis), and share experiences (knowledge translation) [10].	A series of workshops over 14 months to individually and collectively write poetry focused on enhancing public understanding and confronting negative perceptions of disability [33]. The PWLE were shown how to create and perform poetry by two professional performance poets who facilitated the workshops [33]. The facilitators supported PWLE in creating poetry by sharing themes related to images and values associated with the Paralympics and with life as a young disabled person [33]. The PWLE learned how to express themselves and how to creatively communicate their experiences and feelings through poetry [33]. This moved beyond the research question, as the PWLE began conversing about relevant issues, policies, and practices through poetry as the workshops progressed [33]. Knowledge translation has occurred through five live poetry performances [33].
Theatre/improv/film/video [30, 32]	Theatre, improv, film, and video use a dramatic process similar to a focus group where a topic or question is provided to PWLE [10]. The cast is made up of PWLE and the researcher(s), who provide data which is analysed and represented as a performance [10]. Creating the performance, or play building, is similar to qualitative interpretations, but instead of creating themes, vignettes are created instead to represent the findings of the research question [10]. These ABMs can be used as a method of research and to represent and share research findings (knowledge translation) [10]. For example, the process for improv involves PWLE and researchers sharing what they know (data), framing this as improv (interpretation), and presenting it (knowledge translation) [10].	PWLE were engaged in defining serious perinatal complications (the research question), in developing the video script and storyboard focused on using the PWLE's hindsight for the development of the video focused on timelines and strategies believed to be useful for seeking help and navigating the maternity system, and provided feedback on the progress of the video and the finished product [32]. The initial workshops focused on script and storyboard development began with setting ground rules followed by group warm‐up exercises [32]. PWLE then worked individually creating a storyboard and visual timeline of their serious perinatal complication [32]. These were then shared in group discussions [32]. The workshop discussions were audio recorded and transcribed and thematic analysis was used for analysis [32]. An artist supported determining the composition, form and emotional tone of the video [32]. The initial themes, storyboard, and script were shared at the next workshops where verbal and written feedback was solicited [32]. This feedback was incorporated, leading to refined themes, script and storyboard [32]. Finally, the video was created and shared with the PWLE to solicit their feedback on its usefulness [32].
Visual art [28, 30, 31, 37]	Visual art includes all forms of art that are viewed, including painting, sculpting, drawing, collage, and so on [10]. Visual art conveys multiple meanings, as the artist and viewer may interpret the work of art differently [10]. Visual art can be emotional to create or to view [10], as the elements, including colours, texture, tones, light, and topic come together differently for each artist and viewer [64]. Visual art can be used to representing data, either by PWLE creating art or artists creating art based on the experiences shared [10]. Researchers and PWLE can co‐create art to analyse and make sense of the data analyse [10]. Finally, visual art can be created or shared to translate knowledge generated in the research [10].	One study involved PWLE in the research and education priority‐setting [31]. In this study, a photographer took portrait photographs of each PWLE or photographed their hands and the back of heads if they wished to remain anonymous [31]. The photographs were printed and PWLE created visual art of their concerns about their condition, healthcare quality, and safety on their photograph [31]. PWLE were asked to bring an item that summarized their patient experience to the session and this was also photographed [31].
Visual Voices [35, 36]	Visual Voices uses multiple sessions to address a lived‐experience informed research priority [35, 36]. The first session is an introduction to the series of sessions, the process, and the co‐creation of ground rules used to guide future sessions [35, 36]. The second‐fourth sessions involve painting specific to the lived‐experience informed research priority followed by a group critique of the paintings created [35, 36]. The fifth and sixth sessions involve drawing and writing about the lived‐experience informed research priority followed by a group critique of the drawing and writing created [35, 36]. The seventh and final session involves selecting which paintings, drawings, and writing best summarize the ideas generated to be included in the final exhibition and planning the exhibition [35, 36]. The paintings, drawings, and writing are layered to be viewed together, as one final collaged exhibition of the PWLE's experiences, thoughts and feelings [35, 36].	PWLE were engaged in determining the direction of the research and as co‐facilitators/facilitators of the sessions, be it the creation of art or discussions [35, 36]. Over 4–8 workshop sessions, PWLE addressed the research question, places in their communities were they feel safe and unsafe [36]. The workshops followed the format described in this table [36]. Painting occurred on brown craft paper that had been cut into three 5‐foot segments [36]. Paint and brushes were provided for each PWLE and they were encouraged to share paint cups [36]. In the writing and drawing sessions, the PWLE were given white, letter‐size paper and marker and asked to write, draw, or do both based on their comfort [36]. After these sessions, PWLE helped to build the Visual Voice display piece by cutting the dried paintings into 8‐inch vertical strips which were then alternated to form a collage [36]. The writings and drawings were photocopied onto plastic transparencies, taped onto thin plexiglass sheets, and hung in front of the collage [36]. This layering allowed the art and writings to be viewed together as one piece of visual art [36]. All paintings were digitally photographed and writings, drawings, and audio recordings of the group critiques/discussions were entered into qualitative data analysis software [36]. These data sources were iteratively reviewed and thematically analysed guided by PWLE priorities such as ‘nighttime’ and ‘vacant housing’ [36]. PWLE reviewed the preliminary findings, supported interpretation and contextualization, and provided suggestions on revisions to themes [36]. For knowledge translation, a piece of the final collage display was presented in both a community and academic setting [36]. 2‐3 h facilitated group discussions were held over weekly over months [29]. PWLE were provided with digital cameras, training, and feedback on photographs and photovoice skills by a professional photographer familiar with photovoice and working with PWLE after practice photographs were taken [31]. Each PWLE submitted 6‐7 photographs related to the research question [29]. The following questions guided the discussions of the photographs [29]
Photovoice [28, 29, 38]	Photovoice involves providing PWLE with cameras and asking them to photograph their environment related to a research question [10]. They document their environment from their perspective and based on their interpretation of the research question [10]. The photographs are used as sources of data which are discussed in interviews or focus groups to support the sharing of explanations of the photographs, environment, and research question [10]. The photographs can be included in knowledge translation to support an understanding of the experiences of PWLE [10].	1 What does this photograph mean to you? Why is this photo in particular most significant to you? 2 How do you see this photo as a reflection of the issue of supportive long‐term relationships ‐ and one that is relevant to you as a former youth in care in your community? 3 What is the relationship between the content of the photo and how you perceive the community/the world around you? What recommendation for change in your community is associated with this photo?
		Thematic analysis of the photographs and the transcripts of the discussions was performed with the PWLE [29]. Selected photographs and captions were showcased at a photo exhibit event co‐organized by the researchers and the PWLE [29].
Digital storytelling [27]	The PWLE select a story they feel reflects the research question and they, along with the researchers, discuss the stories as a group [27]. Throughout a 2‐3 day workshop, the PWLE write a narrative of their story, record this narrative, and add photos, videos, music, or other multimedia to create a short video that tells their story [27]. After the stories are completed, they are shared in the group setting [27]. Digital stories can also be used for knowledge mobilization, as they are a way to share study findings with patients, healthcare providers, policy makers, and academics [27].	A two‐day digital storytelling workshop format was adapted to incorporate Indigenous elements, such as a land acknowledgement, tobacco offerings, a smudge, talking circles, and a feast [27]. Creating the digital stories involved first choosing the story that they wanted to tell and then writing a 300 to 500 word narrative of approximately [27]. This narrative was then digitally recorded and combined with personal photos and music on iMovie [27]. The creation of digital stories was broken up with three talking circles and the feast [27]. The second workshop ended with a viewing of all the videos [27].
Draw‐write‐tell approach [26]	Draw–write–tell modifies the draw‐write approach, where a drawing activity is used alongside written words produced by the participant [26]. Draw‐write‐tell includes PWLE data analysis, where PWLE interpret their own artwork [26]. This interpretation is recorded and is treated as an equal data source alongside the drawing and writing created [26].	Plain or coloured A4‐sized paper or an iPad will be used, or a collage can be created [26]. The PWLE will be shown 3–4 images that share what research is and why it matters, why engagement in research and healthcare is important, and what a living lab is [26]. The facilitator will show an image and read a brief story about each topic [26]. PWLE will then be asked to complete the story through drawing or collage [26]. The first drawing or collage will related to the question ‘If we were going to create a place to share ideas about research, what would it look like, what would it focus on, and what would it be able to do?’ (the draw component) [26]. Words can be added to the drawing/collage (the write component) [26]. When the drawing/collage is complete, the PWLE will be asked to describe its meaning (the tell component) [26]. Open‐ended questions, prompts, and follow‐up questions will be used to explore all aspects of the drawing/collage [26]. The drawing/collage can be kept by the PWLE as a photograph of the art can be used to support data analysis [26]. The artwork, written, and verbal data will be thematically and content analysed to create a ‘commentary’ with PWLE feedback to inform the development of a survey to develop a living lab [26].
Spoken word [30]	A type of poetry that is not written, but rather is performed [65]. This leads to the focus being not on the visual layout of the poem on a page but on the aesthetics of the sounds created, such as rhyme, repetition, improvisation, and word play [65]. Spoken word can be influenced by music, such as rap, hip‐hop, jazz, rock, blues, and folk music and by storytelling and theatre [65]. Spoken word poems frequently refer to issues of Topics such as social justice, politics, race, and community are commonly associated with spoken word [65].	The 19 PWLE artists were not required to have a formal medical diagnosis of mental illness but rather self‐identified [30]. Workshops were planned with a PWLE advocate and were planned in a way to support including established and emerging artists, ensure the artists contextualized their work through their involvement in discussions, the use of socially inclusive language, and having the show curated by a respected peer [30]. As the PWLE are artists, this project approached the creation of art differently, having the PWLE submit pieces of art or perform spoken word, theatre, or music for the project exhibit rather than co‐creating them in workshop sessions [30]. To ensure the perspectives of PWLE related to their submitted art were also incorporated, they were asked to write about the role of the arts in their recovery, which was also included [30]. This was reported to demonstrate value for their art and to emphasize their artistic identities [30]. The exhibit was part of a larger symposium and was shown and performed on the first night [30]. The PWLE were invited to the symposium, and all of their expenses were covered [30].
Songs, singing, music [28, 30]	Music is defined as arranging sounds in a melodious and rhythmic ‘continuous and unified composition’ [10, p. 129]. It can be instrumental or include lyrics [10]. Music is intrinsically social, as it is part of most cultures [10]. Music can be used as a source of data as it includes how meaning is communicated and it can be used for knowledge translation [10]. Music is thought to be the least accessible ABM so it is the least used [10]. This is because of the perception that one must know how to read music, play instruments, or sing to use music in research [10].	PWLE were involved in the development of methods and outcomes for this study [28]. PWLE were provided with materials for a variety of ABM, including photography, poetry, songs, painting, and drawing [28]. They were asked to create at least one piece of art in any medium in response to the research question [28]. A series of questions were used to generate PWLE narrative descriptions of their artwork in relation to the purpose of the research project [28]
		1 Describe your piece 2 What is happening in your piece? 3 Why did you focus on this subject matter? 4 What does this artwork tell us about your recovery? 5 How can this artwork provide opportunities to support the recovery process?
		The responses were audio‐recorded and transcribed [28].
Photography [28]	Photography involves using a camera to capture an image [10]. This can be done by the researcher or PWLE and the photograph can used as a source of data to provide context to conversations and discussions [10]. Photographs can also be used to translate knowledge, as they can be included in manuscripts or in novel knowledge translation methods [10].	PWLE were involved in the development of methods and outcomes for this study [28]. PWLE were provided with materials for a variety of ABM, including photography, poetry, songs, painting, and drawing [28]. They were asked to create at least one piece of art in any medium in response to the research question [28]. A series of questions were used to generate PWLE narrative descriptions of their artwork in relation to the purpose of the research project [28]
		1 Describe your piece 2 What is happening in your piece? 3 Why did you focus on this subject matter? 4 What does this artwork tell us about your recovery? 5 How can this artwork provide opportunities to support the recovery process?
		The responses were audio‐recorded and transcribed [28].
Cultural amination [34]	Cultural animation was developed and pioneered by New Vic Borderlines (the outreach department of the New Vic Theatre, Newcastle‐under‐Lyme, UK) with the Community Animation & Social Innovation Centre (CASIC) at Keele University [34]. In cultural animation, individual and collective goals are determined and then are met through the ‘everyday experiences of ordinary people and their creative abilities’ [34]. Outputs of cultural animation include songs, poems, short plays, puppets, and transcripts of discussions [34]. Cultural animation workshops include a combination of creative tasks and small group discussions to explore the topic of the research [34]. The workshops begin with group exercises to build trust, develop relationships among participants, and introduce the concept that there are many ways to approach and solve problems [34]. These exercises also begin to break down power differences among PWLE and researchers [34]. The exercises and the art created encourage reflection on the potential for change within PWLE and their communities [34].	Three workshops were conducted to explore the concept of healthy communities [34]. Workshop 1 explored the question ‘What is a healthy community?’ [34] This workshop began with a presentation of the current local health statistics by a retired health system executive, which inspired a conversation about the definitions of healthy and unhealthy [34]. PWLE were then separated into two random groups and asked to create art depicting what ‘a healthy community’ feels, looks, sounds and tastes [34]. The second workshop explored ‘ageing well and dying at home’ [34]. PWLE listened to a recorded story about an older woman and then described their feelings that were evoked [34]. They were then asked to identify how an ideal community for older people would make a difference to their lived experience and to dying in one's own home. The third workshop explored ‘how communities and government could work together to improve health’ by asking PWLE to create a ‘picture of health’ [34]. This was achieved through two activities, using social media to convey their findings to a general audience and a human tableaux presentation to government [34]. The workshops were recorded and transcribed and the transcripts and artwork were analysed concurrent to the workshops using narrative analysis [34].
Zines [40]	Zines are self‐published booklets of visual art such as drawings and collages, along with written art, such as poems [40]. Zines have been historically used as emancipatory tools supporting feminism, antiracism, and far‐right politics [40]. They are often shared through zine libraries, which require an awareness of their existance to access them [40]. The close communities exploring these emanceptory topics share zines to support social justice and advocate for change [40].	Zine‐making workshops were co‐planned with PWLE [40]. Two workshop, each consisting of two 90‐min long sessions, were delivered virtually. The materials required for zine‐making, including paper, colour tapes, stickers, etc, were sent to participants to support accessibility [40]. A PWLE co‐faciltiated the second workshop [40]. During the first session, the history of zines was shared along with the concept that art is meant to share experiences, not convey beauty [40]. Participants were provided time to reflect on what important theme they wanted to express in their zine and who they wished to share their zine with [40]. Participants could continue to work on their zine between workshop sessions or complete it in the second session [40]. All participants were invited to share their zine or to send photoraphs of it, however, sharing was not required [40].

### 4.4 Impact of Arts‐Based Methods on Patient and Care Partner Engagement

While no studies explicitly evaluated the impacts of ABM on PER, there were a variety of perceived impacts of using ABM for PER noted in these articles (Table 1). We perceived the inclusion of the various forms of art provided a deeper understanding of the perspectives of PWLE, as their knowledge was shared directly through their art [28, 31, 34, 35, 36, 39, 40]. These studies included the art created in their articles, allowing readers to experience the art for themselves along with reading the analysis of the project. One of these studies shared a coded piece of visual art as a way to explain the codes created from the data [36]. Another study shared the poems that were created to describe the results of the review, co‐authored by a PWLE [39]. These poems were all translated into other languages [39]. Planning the translation brought the nuance of phrasing into consideration, which led to the inclusion of cultural context informed by lived experience [39].

We also perceived that this inclusion shifted the power in the relationships and the research [28, 29, 31, 34, 35, 36, 37]. This power shift occurred through the ability of PWLE to directly share their lived experiences, with researchers included in a supporting role. One article, which was co‐authored by PWLE, reported the following related to ABM shifting power from researchers to PWLE:

(this method) was necessary for my participation due to its character of *direct democracy* that contrasted with previous experiences…Since the project *equalized power dynamics* to a significant degree, I felt my interests and needs were *valued*…my voice and experiences *contributed to meaningful dialogue* that had an impact on how we governed ourselves and the project itself. My experience of alienation and exploitation, which are present in other projects, was mitigated from the *consensus and dialogue‐driven process.* [29] (emphasis ours)

This PWLE recognized the benefits of using ABM for PER in reducing the power imbalances that inherently exist in research [29]. While PER begins to shift power from researchers to PWLE through the inclusion of PWLE as peer or co‐researchers researching themselves [29], this power shift can be furthered with the use of empowering research methods such as ABM. These authors noted that ABMs empower PWLEs to share their experiences in a meaningful way rather than having their experiences interpreted and told by others [29]. The PWLE authors use the term ‘voyeuristic’ when describing researchers taking photographs, which highlights the power imbalances that can exist between researchers and PWLE when research or engagement methods are not perceived as empowering [29]. Researchers may continue to identify themes and select quotations to include, limiting this shift in power. Including PWLE on the authorship team as this team did [29], extends this power shift. As co‐authors, PWLE contributed to the crafting of the paper and impacted data analysis and presentation [29].

Similarly, the use of ABM for PER was perceived to reduce the perception of tokenism that can plague PER [30]. These authors perceived that the use of ABM shifted the engagement from a checkbox towards empowering PWLE and using the research to meaningfully share the art created [30]. Including artists from the research in the art symposia that shared research findings was noted by PWLE as valuing their contributions by empowering PWLE and showcasing their perspectives [30].

One group of authors perceived that digital storytelling, their ABM, was a ﻿decolonizing method that could be used to engage with and learn from PWLE [27]. Digital storytelling was argued to be decolonizing as it supported enacting cultural humility and provided PWLE with the ability to creatively and culturally appropriately share their complex experiences [27].

Using ABM for PER allowed PWLE to share aesthetic knowledge, that which cannot be put into words: emotions, feelings, and relationships [32, 33, 38]. ABM allowed for creativity and self‐reflection on experiences and the ability to express these in ways ‘not confined by words alone’ [38]. One group of authors used a workshop focused on sharing emotions [33]. They reflected on their ability to share emotional experiences using emotive terms such as *express, portray*, and *explain* [33]. Sharing emotions can come at a price, however. Although PWLE in another article recognized the value of using their experiential knowledge to help others, they paid an emotional toll that they share through the theme of ‘the emotional cost of involvement’ [32]. Sharing their experiential knowledge through ABM ‘revived’, ‘painful’, ‘distressing’, and ‘upsetting’ emotions [32]. These PWLEs believed that it was a toll worth paying, however. This is because sharing their emotions was ‘cathartic’ and they believed that ‘if it saves one baby it's worth it’ [32].

## 5 Discussion

This review demonstrated that the number of studies employing ABM for PER are increasing over time. The use of ABM in PER is more common in Canada and the UK than in other countries. There are a variety of ABMs used for PER and more than one ABM can be used for PER in a single study. ABMs can be integrated into projects with diverse objectives for PER and where PER is planned for a variety of stages of research. The integration of ABMs for PER research can be decolonizing [27], enriches the results shared [28, 31, 34, 35, 36, 37], balances power between researchers and PWLE [29, 37], and helps to convey the nonverbal, which PWLE value [32].

### 5.1 Growth in Arts‐Based Research Methods for Patient and Care Partner Engagement Over Time

The growth in healthcare research using ABM for PER over time may relate to the support from national funding bodies. PER has gained national and international attention and support over the last decade. While the James Lind Alliance has existed in the UK since 2004, they note that awareness of PER was limited until 2010 [42]. In 2011, CIHR shared their Strategy for Patient‐Oriented Research [43]. In the same timeframe, there has been growth in the use of ABM throughout the healthcare research process [10, 14]. This timeline aligns with the rise of alternative theoretical underpinnings for research, including critical social theories, which value more diverse epistemologies than traditional biomedical theories [44, 45]. Socially constructed knowledge is a tenant of critical social theories [45] and ABM [46], so these theories provide healthcare researchers with the opportunity to use ABM to elicit embodied, emotive knowledge [10] previously missing from the healthcare literature.

### 5.2 Geographic Ties to Arts‐Based Research Methods for Patient and Care Partner Engagement

The number of studies conducted in Canada using ABM for PER is unsurprising. PER is a priority for CIHR, supported through its Strategy for Patient‐Oriented Research [43]. A set of five broad initiatives was shared by CIHR in 2011 with the goal of advancing PER in Canadian research [43]. Since this time, CIHR has invested in growing the pool of researchers engaged in PER, created ﻿specialized PER service centres, and supported the creation of ﻿PER networks as three ways of supporting PER [43]. CIHR also supports ABMs, although in a less formal way not through an initiative or priority area. For example, CIHR supports the use of ABMs for knowledge translation [9]. In a CIHR Institute of Gender and Health publication, ABM was highlighted and tips for integrating ABM into research were shared [47]. The author is a Canada Research Chair, further suggesting that CIHR values ABM.

The six studies conducted in the UK likely reflect the support of The National Institute for Health and Care Research for the James Lind Alliance [42]. The James Lind Alliance brings together PWLE and clinicians in Priority Setting Partnerships which can be used by funding bodies to ensure alignment of funding with the priorities of those impacted by research [42]. As well, UK Research and Innovation supports ABM and PER through research grants, for example, this climate change funding opportunity [48]. The number of studies involving using ABM for PER from Canada and the UK suggests that explicit support from national funding bodies for both ABM and PER encourages researchers to engage PWLE using ABM.

### 5.3 Arts‐Based Research Methods Synergy With Patient and Care Partner Engagement

PER using ABM can occur with a variety of objectives, at many different levels, and at many different stages of research. The use of ABM for PER was noted to reduce tokenism [30] which is a known issue in PER [49, 50, 51]. The variety of levels and stages at which PWLE can be engaged suggest that the ABMs employed need to be tailored to the specific project. Of the variety of ABMs available [10], a wide array has been used for PER, from written genres, such as poetry [28, 33, 34, 39], to performative genres, such as film and theatre [30, 32], to visual art [28, 30, 31, 34]. Narrative enquiry is an ABM that should be considered for use with PER. The goal of this collaborative process is to create a story from the experiences PWLE shared [10]. Similar to fiction literature, narrative enquiry cumulates in a story where multiple viewpoints are shared in an aesthetic way [10]. Rather than the more commonly used thematic analysis [26, 29, 31, 32], narrative enquiry applied to group discussions may allow for exploration and a richer description of the experiences shared [10].

Similar to the use of ABM for knowledge translation [46], the use of ABM for PER led to the ability to share experiential, emotional, and aesthetic knowledge [32]. Sharing aesthetic knowledge was noted to lead to discomfort among PWLE in one study [32]. This should be planned for when designing studies using ABM for PER to ensure appropriate support is available for PWLE who become distressed. ABM may also be used to support PWLE, as there is a long history of using the arts for therapies and healing [10]. By diving into an exploration of emotions, identity, and reflection and then being empowered to co‐create meaning, using ABM for PER follows the tenets of arts therapies [10].

ABMs can shift power from the researcher to PWLE, as ABMs are inherently participatory [10, 18]. Sharing the results of studies co‐created with PWLE using the art created is one way that this power dynamic shifts [34]. Stigmatized topics, such as mental health and disability, are approachable through the use of ABM for PER [52] due to their participatory nature and shifting of power [28, 31, 34]. This is because ABM can empower PWLE to share their experiences in a manner of their choosing rather than having their experiences interpreted and their stories told by others [29]. Similarly, topics that are inherently more emotional and complex can be approached by engaging in a non‐verbal, aesthetic exploration [18]. The meaning contained in the art can be visually communicated and cognitively, viscerally, and sensorially felt, thereby acting as a catalyst in the co‐creation of knowledge [18, 53]. Using ABM for PER also opens up the figurative door to engaging youth and older adults, who may not have the words to describe their experiences [18]. As such, using ABM for PER gives voice to those who would otherwise be silenced or ignored.

#### 5.3.1 Epistemological Synergy

The biomedical worldview dominated health research until the last twenty years, when knowledge is available to be discovered through objective research methods [54, 55]. This worldview does not value subjective [54, 55] nor aesthetic knowledge [10] and can be viewed as oppressive [56]. An alternative worldview that values that an experience may be interpreted differently by people of different backgrounds, socioeconomic statuses, or genders is required to include the context provided by PWLE as a source of knowledge [54, 55]. To guide PER using ABM, this alternative worldview must also value that these experiences can be shared aesthetically.

A pragmatic worldview moves away from the pursuit of objective truth and advocates using the most appropriate research methods to produce logical, rational, and relevant research findings [57, 58]. Through this worldview, the research question drives the choice of design and methodology [57, 58]. The separation of the question from the assumptions of a biomedical worldview is key to a pragmatic worldview [58]. Rather than being defined by how one knows and whether reality is subjective or objective, a pragmatic worldview is defined by the requirement to conduct purpose‐driven research [58].

There are several key points of a pragmatic worldview that divulge the shift from studying people seen in a biomedical worldview to engaging them in research through ABM. The focus of research guided by a pragmatic worldview on producing relevant research findings [57, 58] suggests there must be an understanding of what is and is not relevant to the population. The belief that shared experience can lead to shared understanding [57] and that knowledge is derived from action [58] suggests that the views of PWLE are required to understand a health issue. The experiences of a researcher or healthcare provider of a specific health issue are very different from patient experiences. For research to truly be directed at improving patient health outcomes, one must understand all experiences and, thus, engage PWLE in research [57, 58]. PWLE can be engaged in novel, arts‐based ways to inform projects and share their lived experiential knowledge, including their aesthetic knowledge. By challenging and providing an alternative perspective to the limitations of what constitutes knowledge by the biomedical model, ABMs become anti‐oppressive and emancipatory [39, 56].

### 5.4 Patient and Care Partner Engagement and Participatory Action Research

Four of the studies included were described as participatory action research (PAR) rather than PER [28, 29, 36, 37]. The history of PER is rooted in PAR, such as the disability and HIV/AIDS movements that coined the phrase ‘nothing about us without us’ [59, 60]. PAR is a social sciences research approach that is collaborative and contextual with the goal of social change [61, 62]. It challenges power dynamics inherent to research by removing the hierarchy of the roles of ‘researcher’ and ‘participant’, placing the two as equal collaborators with unique and important perspectives [61, 62]. PAR is contextual as it depends on knowledge embedded in a particular place and time [61, 62]. To enact change, knowledge keepers embedded in the setting actively participate and collaborate with researchers and co‐create knowledge owned by the entire team [61, 62]. This approach moved from social to health research when the CIHR explored bridging the know‐do gap and moving research findings into clinical practice [43]. Feedback from multiple stakeholders specified that research needed to include the perspectives of PWLE and consider the local contexts of healthcare delivery [43]. The CIHR Strategy for Patient‐Oriented Research to address the know‐do gap does not state it is guided by PAR; however, it is evident that the CIHR recognized the power of PAR in considering both the lived experience and local contexts [43]. This expanded the engagement of PWLE to the mainstream of health research.

### 5.5 Implications for Future Research

Despite the low volume of studies using ABM for PER, this review highlights the importance of expanding the operationalization of PER to include ABM, as well as reporting the impacts of the methods used for PER. This review also highlights the variety of ABMs available for PER and the importance of explaining why the specific ABM was chosen. There are ABMs that could be used for PER, such as body mapping, that were not found in the existing literature. Research is required to understand the operationalization and utility of these ABMs for PER. Current evaluations of the impacts of PER focus on the impacts on the research process [63]. Research is required to explore the impacts of the complex experiences shared by PWLE through ABM beyond the research process. For example, how complex experiences can be used to inform the development of a complex intervention and the impacts of sharing complex experiences on both PWLE and researchers. Finally, research is required to understand the utility of aesthetic experiences shared through ABM for PER in the creation of knowledge and co‐development of interventions.

### 5.6 Limitations

This narrative review has several limitations. As narrative reviews do not include a quality assessment, the quality of the studies included in this review was not assessed [22]. Also, no grey literature was included. Finally, only one reviewer completed screening.

## 6 Conclusion

This review demonstrates that the use of ABM for PER is becoming increasingly common in countries where ABM and PER are supported by national funding bodies. As a viable anti‐oppressive method, ABMs for PER can be meaningfully utilized with vulnerable populations and to unpack stigmatized topics. A variety of ABMs can be used across many levels and stages of PER. When ABMs are used for PER, there is a positive impact on the study and on PWLE. For researchers engaging PWLE, using ABMs to elicit aesthetic knowledge [11] equalizes power among team members [19, 34]. Expanding the tools for PER and improving collaboration and co‐creation of knowledge will positively impact the healthcare system and those receiving care.

## Author Contributions

**Emily K. Phillips:** conceptualization, writing–original draft, writing–review and editing, formal analysis, data curation. **Anna M. Chudyk:** writing–review and editing, visualization. **Caroline Monnin:** methodology, writing–review and editing, data curation. **Annette S. H. Schultz:** writing–review and editing, supervision. **Rakesh C. Arora:** supervision, writing–review and editing. **Todd A. Duhamel:** writing–review and editing, supervision. **Sheila O'Keefe‐McCarthy:** supervision, resources, conceptualization, writing–review and editing, methodology.

## Ethics Statement

The authors have nothing to report.

## Consent

No patients were required to provide consent for this review.

## Conflicts of Interest

The authors declare no conflicts of interest.

## Permission to Reproduce Material From Other Sources

No materials are being reproduced from other sources.

## Data Availability

The data that support the findings of this study are available from the corresponding author upon reasonable request.
